# Unlocking dimensions of social capital in the prison setting

**DOI:** 10.1186/s40352-016-0040-z

**Published:** 2016-08-22

**Authors:** Lise Lafferty, Carla Treloar, Tony Butler, Jill Guthrie, Georgina M. Chambers

**Affiliations:** 1 The Kirby Institute, UNSW Australia, Level 6, Wallace Wurth, Sydney, NSW 2052 Australia; 2 Centre for Social Research in Health, UNSW Australia, Sydney, Australia; 3 National Centre for Epidemiology and Population Health, The Australian National University, Sydney, Australia; 4 National Perinatal Epidemiology and Statistics Unit, UNSW Australia, Sydney, Australia

**Keywords:** Social capital, Prisons, Inmates, Hepatitis C, Qualitative research

## Abstract

**Background:**

Social capital has been shown to be a valuable resource for improving health outcomes. However, it has received little attention in the prison setting. Dimensions of social capital in mainstream society are likely to function differently among inmates in prison. This study seeks to identify and understand social capital dimensions among incarcerated men living with hepatitis C.

**Methods:**

In-depth interviews were conducted across three correctional centres in New South Wales with 30 male inmates living with hepatitis C. Interviews were transcribed then thematically coded and analysed.

**Results:**

There were differences in the access and utility of social capital dimensions in prison focusing specifically on trust and safety, informal and formal networks, agency, and civic engagement.

**Conclusions:**

Dimensions of social capital do not necessarily translate into prison. An inmate’s social capital may foster greater treatment uptake relating to health and rehabilitative programs during their incarceration.

## Background

Social capital theory has gained increased attention across a number of disciplines including health, economics, governance, and public policy over the past several decades (Shortt, 2004). Social capital can be described as an individual’s accumulation of social resources, inclusive of social networks, social cohesion, and social support (Almedom, 2005). It enables a person to achieve more than would be possible if acting independently. Social capital is “made up of social obligations” or “connections” (Bourdieu, 1986:243). In essence, social capital is “a resource for persons” (Coleman, 1988).

Social capital is a valuable social resource that encompasses a variety of dimensions and has the ability to improve an individual’s social and emotional wellbeing as well as their physical health (Rocco & Suhrcke, 2012). However, in the prison setting, the usual avenues for building and accessing social capital are often not available to inmates. Similarly, incentives for developing social capital in prison may be different than social capital drivers within the community. In the prison setting, social dynamics and human needs may be re-prioritised differently than if they occurred in general society. It is in this way that social capital, or dimensions of social capital, may be experienced differently within the prison setting when compared with benefits of social capital within the community.

Understanding how social capital may impact on health in this population group requires an understanding of prisoner demographics and issues. Persons entering into or cycling through the criminal justice system report greater burden of communicable and noncommunicable diseases than those in the general community (WHO, 2014). Prisoners often report low rates of educational attainment and employment, along with high rates of mental health disorders (AIHW, 2012). In addition to these socio-economic disparities, the inmate population reports higher levels of drug use and, in particular, injecting drug use (Butler et al. 2015) bringing specific risk of exposure to blood-borne viruses (such as hepatitis B, hepatitis C, and HIV). For example, prison inmates worldwide have an HCV-antibody prevalence of approximately 26 % (meaning over a quarter of inmates carry the virus) (Larney et al. 2013), compared with a 1.6 % prevalence in the global general population (WHO, 2016). These combined socio-economic and health factors suggest a different landscape of social capital for prisoners, where those in society are likely to have more favourable socio-economic and health indicators than those in custody. Prison is an opportune time to address public health concerns among this population group, such as initiation of treatment, patient engagement, and identification of acute and chronic health conditions (Glaser & Greifinger, 1993).

In mainstream society, that is, outside of prison, social capital commonly encompasses a number of dimensions including: trust and safety, reciprocity, formal networks (such as group participation in sporting groups) and informal networks (such as friends and family), civic engagement (often measured through participation in civic associations such as volunteering), and agency (a person’s inner drive and self-motivation as accessed through their social networks and supports) (Onyx & Bullen, 2000; Putnam, 2000; Western et al. 2005).

Social capital may provide networks for gaining employment (Moerbeek & Flap, 2008), or neighbourly support such as informal childcare or transportation assistance (Kawachi, 1999). Social capital thus operates to provide greater ‘resources’ among social networks, which may contribute to improved or enhanced quality of life. Accessing health care has also been associated with social capital (Pilkington, 2002), through information dispersal (Coleman, 1988) as well as provision of resources necessary in accessing healthcare (such as transportation to appointments, childcare, and home support during recovery). Putnam (2000) provides an example of social capital directly improving a person’s health whereby a member of a bowling league in need of a kidney transplant found a donor within his league.

Within the context of existing social capital measures, trust has been asked as “do you agree that most people can be trusted” (Onyx & Bullen, 2000). More specifically, trust is assessed as a person’s ability to trust family members, community members, government officials, and others in positions of authority (Narayan & Cassidy, 2001). In the prison setting, low levels of trust have been found to impede upon relationship development between inmates and staff (Harvey, 2012), a barrier in the construction of social capital.

Informal networks commonly refers to friends and family, including partners, while formal networks refers to those social networks within formal structures such as employment, education, and program participation (Putnam, 1998). For those inside prison, informal networks become separated: informal networks with those inside prison and those that remain outside. Connections with formal networks often dissolve upon incarceration as connections to employment and other formal programs on the outside are severed and new formal networks need to be established inside the prison. Compounding this is that inmates are often relocated to other prisons during their sentence (Bretaña et al. 2015); building effective informal and formal networks on the inside while trying to maintain existing informal networks on the outside creates numerous challenges. Informal networks on the inside rely on other inmates and are thus quite different than those networks of support on the outside and likely serve a different purpose within the inmate’s life. Likewise, formal networks on the inside are quite different as inmates have less choice around both access and type of employment, education, and rehabilitative opportunities and, for a multitude of reasons, may be more resistant to engage. All of these experiences and dynamics contribute to the complexities of utilising the social resources provided by informal and formal networks whilst incarcerated. Informal and formal networks can be beneficial to a person’s health through provision of care and social supports that may be required during times of chronic or temporary poor health. However, in prison, these benefits may be compromised due to restrictions of access to networks.

Agency is the “capacity to take the initiative, to be proactive” (Onyx et al. 2007:217). The process of actioning or expressing agency requires the ability to have control over and input into the decisions one makes about aspects of their life (Gordon, 2005). For agency to be viable, “actors must be aware they possess agency and believe they can make a difference through exercising it” (Ling & Dale, 2014:4–5). In essence, agency is a mechanism by which individuals are able to action personal change. However, barriers and the complexities of prison life often prohibit an individual’s capacity to ‘take initiative’ and ‘to be proactive’, particularly within the constraints of the prison environment. Although inmates are able to participate in group activities, such as bible study (or other religious group activity), training and exercise, or informal activities in their wing, prison is itself an authoritarian setting with limited opportunity for self-initiation or action (when compared with the general community). Opportunities for self-improvement are often guided by the court system or parole officer, with limited capacity for inmate’s to initiate self-change.

Civic engagement refers to active participation in civic associations (Condeluci et al. 2008; Putnam, 2000). Portes (1998:18) explains that “civicness” is commonly “equated with the level of associational involvement and participatory behaviour in a community and is measured by indicators such as newspaper reading, membership of voluntary associations, and expressions of trust in political authorities”. While newspaper reading may be a feasible indicator for use in prison, more obvious implications or barriers to civic engagement on the inside are conjured within heightened levels of distrust of authorities. Scope to participate in civic engagement on the inside is likely to be limited.

Experiences of social capital among prisoners are complex and interesting because inmates’ scope to ‘freely’ build social capital is confined by the physical and social perimeters and regulations of the prison complex. Consequently, it may be that a number of dimensions of social capital may ‘transfer’ to the prison setting, but not all are ‘translatable’ – that is, while trust and safety (and other dimensions) may be a necessary component of social capital both outside and inside prison, trust and safety may look different, or be valued differently, in the prison environment than in mainstream society. Other dimensions, such as civic engagement, may not be transferable or translatable to the prison setting.

Previous research has identified a gap in social capital literature within the prison setting (Lafferty et al. 2015). This paper aims to address this gap by identifying how specific dimensions of social capital are experienced in the prison environment, looking closely at the transferability and translation of trust and safety, informal networks, formal networks, agency, civic engagement, and implications of these social capital dimensions on health within this population.

## Methods

This paper specifically aims to understand social capital among inmates living with hepatitis C. HCV is highly prevalent among the global prison population, with an estimated 26 % of prisoners worldwide being HCV-antibody positive (Larney et al. 2013). The general population has a prevalence of about 1.6 % (WHO, 2016). Given this high rate of prevalence, inmates living with HCV represent a significant minority of people within the prison setting in which to examine social capital.

Data for this paper comes from a larger study looking at hepatitis C (HCV) treatment uptake among male prisoners and its correlation with social capital. Three New South Wales (NSW) correctional centres were purposely selected for use within this phase of the research in efforts to capture the social capital dimensions existing in prisons across minimum, medium and maximum security classifications as well as urban and regional settings. The three prisons, one based in Sydney and two in regional NSW, were chosen for this study because a Nurse Led Model of Care HCV treatment service delivery (Lloyd et al. 2013) was operating at each of these correctional centres. A total of 30 male participants were recruited. Eligibility criteria required that all participants be male, be aged 18 years or older, screened positive for HCV, and incarcerated in one of the participating prisons. Potential participants were identified by nurses at each of the sites during clinic visits and were invited to participate in the study. Interested participants provided verbal consent to the nurse who then provided the participant’s identification number to the interviewer (LL) to protect confidentiality of participants. The interviewer attended the prison and met with potential participants. The interviewer reviewed the participant information sheet and received written consent from participants prior to commencing interviews. Interviews were conducted within the health clinics at each of the participating correctional centres. Participants received AUD$10 in their inmate bank account as remuneration for their time in completing the interview. Interview times ranged between 13 and 79 min with a median time of 37 min.

The interview schedule covered dimensions commonly included in general social capital instruments. These ten dimensions were: informal networks (including family and friends), formal networks (including employment, education, religion, and rehabilitative program participation), trust and safety (including inmates/peers and staff), civic engagement, communication, community, reciprocity, culture, and health and wellbeing (including items about discussing HCV and HCV treatment with others). The interview schedule was reviewed for both content and clarity by experts in the field, a research reference group, and a former inmate.

Interviews were conducted using appreciative inquiry, an interview method that allows self-reflection of the positive social capital available to and accessed by the participant. Appreciative inquiry was selected for its ability to draw out sources of social capital within the prison setting (Liebling et al. 1999), particularly focusing on beneficial gains from social resources rather than negative experiences associated with the prison setting. Demographic data were collected from participants at the completion of the interviews.

The interviewer wrote field notes throughout data collection, noting new conceptualisations of social capital and/or experiences of social capital in unanticipated ways. These field notes were communicated to the other authors throughout the data collection process. Following each day of data collection, the interviewer debriefed with a forensic mental health nurse. These debriefings were completed as part of a safety protocol measure and allowed the interviewer to clarify meanings and develop ways to introduce ideas to explore in future interviews.

Interviews were audio-taped and transcribed verbatim. Transcripts were then checked for accuracy, and de-identified. QSR NVivo 10 was used to organise, code and retrieve data. The data underwent two rounds of analytic coding; memos were recorded during the coding and analysis process. The first round of coding used the pre-identified themes as written into the interview schedule. The second round of coding was completed to code the data into sub-themes emerging throughout the data collection and analysis processes. The second round of coding allowed for identification of inter-relationships between social capital dimensions (Dudwick et al. 2006) and to clarify how sub-themes related to “parent” dimensions, e.g., inmate employment had scope to represent trust and safety (such as employment arrangements featuring or dependent upon trust between staff and inmate) as well as civic engagement and social agency (whereby inmates may have opportunity to articulate and act on preference). Variables which may contribute to an inmate’s social capital, such as age, security classification, overall sentence length, time served and time remaining, and prison culture, will be reported where relevant.

Three Human Research Ethics Committees provided approval for this study (Aboriginal Health & Medical Research Council (986/13); Corrective Services NSW; and Justice Health & Forensic Mental Health Network (G633/13)).

Participants are referred to by pseudonyms to protect anonymity. Their age and security classification have been included. Participants in protective custody are indicated with an asterisk beside their classification.

## Results

Participants included men across a range of HCV treatment stages: those who had never accessed treatment, those who were considering treatment, as well as men who were currently undergoing treatment. Men were recruited across all security classifications including minimum (C), medium (B), and maximum (A) security as well as escapee (E) (reserved for inmates who have attempted or successfully escaped) (CSNSW, 2015). Security classification is based on a person’s crime, sentence length, and previous criminal history (CSNSW, 2015) and are an indication of the privileges and restrictions bestowed upon inmates. Approximately one-third of participants were in protective custody for various reasons.

### Trust & safety

Trust and safety is a challenging dimension of social capital within the confines of prison. Prisoners may have complex issues regarding trust of authority figures, stemming from pre-incarceration as well as past and current incarceration experiences. Likewise, interpersonal trust with other inmates may be inhibited due to concerns of safety and protection and other cultural intricacies specific to prison life. Investing trust in other inmates may leave the individual both emotionally and physically vulnerable. The inmate experience is often a transient one as inmates are transferred between facilities, released to freedom, and some are re-incarcerated. Similarly, trusting staff may be contentious as they represent ‘the system’.

Participants routinely responded with immediate disapproval when asked whether they could trust any of the correctional system staff. It seemed that no personnel category – i.e., welfare officers or other caregiver personnel – were immune from this inherent distrust of ‘them’ by inmates. However, when asked about specific personnel, such as the nurses, the participant’s employer in prison or prison education officers, many inmates were more reflective and considered staff as individuals rather than broadly implicating all staff within the correctional system as not being trustworthy. The way in which staff treated inmates – as a patient, a student, or an employee – and not relying on stereotypes such as “crims”, were indicative of whether an inmate could then trust an individual member of staff.

Yeah, because [education officers] don’t treat you like the officers treat ya. They treat you like you’re a student, not an inmate. [*What about the nurses?*] Yeah, they’re…it’s same thing. They don’t treat you like you’re an inmate, they just treat you like you’re a patient. (John, 22, C)

The one that’s given us the job, he’s not a bad officer and that. [*What makes him not a bad officer?*] Oh he just, he talks to you like a person, not like you’re a crim, or anything. He’s pretty easy going. (Lee, 33, B*)

Trust and safety are commonly interlinked as a singular dimension of social capital within the literature (Onyx & Bullen, 2000; Trewin, 2006). This unification is reflected in many of the comments made by participants relating to trust and other inmates. Safety appeared to be associated with protection – whether this was in the form of physical protection or ‘keeping an eye out’ (referring to a range of illicit acts) or more basic matters, such as trusting a cellmate with one’s possessions. Other literature links trust and reciprocity as a dual-dimension (Daly & Silver, 2008). This inter-connection is also highlighted within participants’ comments whereby reciprocity was closely associated with sentiments of trust among inmates; a number of participants described reciprocal relationships as a determinant of trust.

[*Is there anyone here that you trust?*] With some things, but not, not everything. [*Not full trust?*] Yeah. [*Okay. So like what’s something you might trust someone with?*] Um…like your cellmate, they don’t steal your stuff. Um…you sort of feel safe in the cell with them, you’re comfortable. (Wayne, 32, C*)

Yeah trust [my cellmate] in everything. Not taking my stuff, discuss personal stuff outside, you know, me daughters and me kids and me missus and all that. […] Just normal, a good mate. (Liam, 49, C*)

The trustworthy ones I met through jail. [*What makes them trustworthy?*] I don’t know, probably just…you just know, like…you trust them, like they fucking, like they’re the first one to have your back if there’s con- they’ll stand by you if there’s. […] Just, you know, you stand by them, they help you out. (Blake, 26, C)

Trust among inmates had meaningful and diverse understandings and connotations. In some instances, as shown above, trust came down to material possessions. In other instances, it was imperative to one’s security on the inside that weakness not be shown, as participants perceived that any vulnerability would be exploited by other inmates.

You can’t, I don’t feel I can trust people here, you know. There just, there’s always, even in jail in general it feels like everyone’s trying to…there’s an angle for something, you know? (Tristan, 23, A)

Because of this place here, like, it’s like, like I said, it just turns you – it makes you into someone where you’ve shown, like you can’t really have weakness. [*Yeah. So you have to be quite guarded.*] Yeah, you can’t, because, what I know about you is, okay, if I find something, I’ll exploit it. Like, not, like I won’t exploit it because I don’t think…but other people would exploit it. [*Yeah. People look for a weakness and then they try and take advantage of that.*] Exactly. So that’s why a lot of people, they don’t bother sharing and opening up. (Adam, 36, E)

Some experiences of trust and safety were found to be more applicable at one prison than others and may be evidence of diverse prison cultures. All the participants who discussed being guarded, or refraining from showing any signs of weakness, were all incarcerated at the same prison, had long prison sentences (9 years or greater), and were currently classified to maximum security. This interconnection of trust and self-protection did not emerge in either of the other two correctional centres in this study where classifications were generally lower and average age of participants was younger.

Several participants indicated a distinction between “friends” in prison and “other inmates”. Making connections with other inmates that would span beyond the prison wall appeared rare; this tended to be the distinction for many participants in their description of trusting other inmates.

I don’t think I’d give them my number and that and call them when I get out. Like me girl’s contact information or anything like that, like where I’ll be going. […] Wouldn’t do that. (Keith, 38, classification not recorded*)

Friends, I use, as a different word. An associate, or you know. Because when those people get out, they don’t care about ya. You know? So it doesn’t…it’s only a friendship while you’re in here. You know, so yeah. They’re not what I’d call…they’re friends, they’re friendly people, but… [*So, like friends on the inside but, friends on the inside doesn’t mean friends on the outside?*] No it’s not going to happen outside. It’s not going to happen outside yeah. Yeah, it’s different. A lot of people in here are different in here to what they are outside too. (Charlie, 49, classification not recorded*)

Trust in prison, whether between inmates or between inmates and staff, had a range of complexities that seemed specific to the prison environment. On the inside, trust in professionals was rooted in the treatment of the individual as notions of trust between inmates and personnel seemed to be intertwined with dignity; that being treated “as a person”, rather than “a crim”, was a central feature of whether an inmate felt they could trust individual staff members. Personal possessions were identified as a source of trust and safety among inmates. However, trust between inmates was often confined to the prison setting and did not extend beyond the prison’s walls – trusted relationships on the inside did not readily translate to friendships on the outside. Notions of trust and safety may be prison-specific with cultural features defining sub-elements of trust and safety that are not translatable to all prisons.

### Informal networks

Inmates may find themselves as having two very distinct informal social networks – those inside prison and those on the outside. Informal networks on the inside may contribute to an inmate’s social capital through social connections as well as someone to empathise with their situation and circumstances whilst informal networks on the outside may perform numerous functions for the wellbeing of inmates through connection and support.

The process of accessing employment while in prison was, for many participants, indicative of multiple dimensions of social capital including informal networks, trust and safety, and reciprocity. Personal connections appeared to be as equally important on the inside for accessing employment as they are for the general population on the outside.

[*How did you get the job as sweeper?*] Ah through one of me mates. He was a sweeper, and they needed another sweeper, so he got me a job. [*So he put your name up for it?*] Yeah. I asked him to. Yeah he said, if a job comes up, put my name on it. And I got it straight away. (John, 22, C)

I just came in, there was a couple of other guys there, and they said, come and work over here. So I started working over there. (James, 44, C)

Some participants reported having connections with other inmates that were imperative to their wellbeing on the inside, particularly as they were isolated from their regular support networks on the outside.

I find that you have to speak to someone, or it’ll drive you mad. You know? Because if you don’t, you start jumping to conclusions. It’s good to hear, like, the way they interpret it, like, I explain the situation and I say to them, ‘alright, how would you take this if this was you?’ And if they can empathise they’ll tell me what they feel. And I’ll go from there. (Adam, 36, E)

And they keep me focused too, like I’ve been having, experiencing problems with the girl at the moment, and my mate that I was telling you about now, he’s doing a long time… […] And said, “get her out of your head mate, it’s going to help ya”. (Gary, 31, A)

These informal networks were also shown to be reciprocal, with many participants providing social and emotional support to other inmates.

Yeah like, just to try and give him a different perspective, you know? And I mean…and yeah…it’s hard. He’s got a… […] If he’s never experienced the other side, I guess it’s hard. You know what I mean? (Matt, 37, E)

Yeah, have a cellmate. Been with him since [another prison]. About a month now we’ve been together all the way through. Still the same cell-y. This is his first time, so I was sort of showing him, like, don’t, don’t worry, don’t stress or nothing. (Damien, age not recorded, A)

Families and friends on the outside were often highly valued sources of social support for inmates. The support provided from loved ones in the community ranged from having someone believe in them, the provision of legal and other support pertaining to their sentence, and fiscal support. Participants were able to maintain contact with those on the outside through visitation, phone calls and letter writing. These connections were mostly with family and partners (few participants reported maintaining contact with friends on the outside). The social support from these connections appeared to be invaluable for many of the participants.

[*Do you write to* [*your dad*]?] All the time. All the time. […] I just jot down a few lines, and say, “hey mate, how you going”, you know? “I’m doing alright. Sorry that I’m writing to you from this place, but…” […] They accept me for who I am. (Keith, 38, classification not recorded*)

My family is here for me. […] My sister, she’s real supportive of me. She talks to my solicitor and that for me. (Damien, age not recorded, A)

A number of inmates talked about the importance of having money deposited into their accounts from family, friends, and their “support group” on the outside - “your people”. These provided a critical lifeline in prison and were able to greatly increase the quality of the prisoner’s life while they served their time, as well as covering the costs of phone calls enabling prisoners to keep in regular contact with family and friends on the outside. Although questions relating to financial support were not in the interview schedule, a third of participants (*n* = 10) discussed receiving money into their account from sources outside. It was predominantly family members who provided weekly or fortnightly deposits, as well as partners and occasionally children and friends.

You get paid $23 a week. That doesn’t even cover, if you smoke, that doesn’t even cover a pack of smokes. So um…I don’t rely on that at all. My money comes from the outside. Like people send me money, you know? [*Okay. So who sends you money?*] Just, like my support group. You know? Family, friends, whatever, you know? (Tristan, 23, A)

It’s up to your people to put money in your account. If you don’t have people out there that’ll put money in your account every week, the only way [inmates] survive… (Byron, 35, A)

Me sister’s going to come down and take me out for the day. […] She’s always ringing up the parole officer to see what I need to do and that. […] And she helps me financially, you know? (Chris, 53, C*)

Informal networks, both inside and outside, seem to have the capacity to improve an inmate’s quality of life. Interestingly, participants’ social connections, both in prison and those outside, often resulted in increased fiscal capital for participants. Informal networks on the inside may lead to better employment opportunities, while informal networks on the outside may provide cash deposits to inmate accounts. Additionally, the social supports provided by informal networks on the inside were shown to, at times, reduce inmates’ concerns about their informal networks on the outside.

### Formal networks

People who cycle through the prison system generally have complex lifestyles and are often connected to multiple social support organisations on the outside. They may have connections with counselling, drug and alcohol services, employment support programs, and a range of other services aimed to improve their outcomes (such as socio-economic, wellbeing and recidivism measures). The decision or motivation to access these services may be a personal choice, court-mandated, or a combination of reasons. Once the person is incarcerated, their connection with these services is severed without much (or any) communication from the service user/prisoner.

The social capital effect of formal networks in prison was unclear. At one prison, where inmates were more guarded (see ‘Trust and Safety’ above) for fear of having weaknesses exploited, only half of the participants reported being employed and none of the participants at this study site were enrolled in any educational or rehabilitative programs. At another prison (in which all participants were in protective custody), over half of the participants were employed at the time of the interview, approximately a third of participants were enrolled in courses and another third were participating in rehabilitative programs. A number of participants reported benefits of accessing education whilst incarcerated – it provided some value to the time inside, turning prison into an investment in self.

Yeah I’m doing like a Year 10 certificate, and then I want to do my Year 12. […] So I’ve got something to get out with. (Mike, 29, C*)

Why not educate yourself? They call this a rehabilitation centre. Why not try and better yourself while you’re in here? (Gary, 31, A)

Accessing education and participating in either employment or rehabilitative programs, suggested expression of agency. However, the benefit or value of this dimension of social capital was not easily translated to the prison setting. Participants (all living with HCV) did not report involvement with programs targeted at people who inject drugs or those living with HCV – programs which would likely provide more identifiable social capital benefits to this population group.

### Agency

Agency is one of the more obvious dimensions of social capital that may not be translatable or transferable to the prison setting. Prison is, through its very purpose, an environment of limited agency; where capacity to make decisions about one’s own life is severely restricted. However, those accommodated inside correctional institutions can express agency through decisions pertaining to oneself and, sometimes, decisions impacting others. A number of participants reported areas in which they were able to take action and make decisions which impacted their lives – both in prison and in preparation for their release.

Post-release planning was an integral feature of change among participants. Post-release planning was one of the most critical opportunities for evidencing drive, motivation, determination, and ambition. For some, this centred on re-connecting with their children, or familial commitments to those who cared for and looked after them. Often, elements of reciprocity were grounded in agency.

But as you get older you realise what’s important to you. You know? And like, for me, it’s just my, to get out of jail while my grandmother is still alive. That’s why I’ve pulled up, you know? […] I’m trying to do something for her this time, instead of always being selfish. It was always about me. You know? So I’m trying to do what’s right for her. (Adam, 36, E)

You know, I’m not up to my old tricks like I used to do when I was younger and that, in jail. […] Bashing, robbing, you know what I mean? But um…it’s pretty, only because of my daughters, you know? […] I don’t want to lose them ever again. […] They’re my life. I’ve got to be out there looking after them. (Pete, 40, B)

I don’t want to be that bad person anymore. I don’t want to keep coming back to this place. I don’t want to be doing drugs. I just want to get my kids back, and just be a father. (Ben, 27, C)

It was anticipated that post-release planning would be context-specific and related to factors such as sentence length, proximity to release, and/or security classification. However, these patterns were not evident as participants who articulated post-release plans ranged in overall sentence length, proximity to release, as well as security classification.

As noted above, all participants in the study were hepatitis C (HCV) positive. Participants included both those currently undergoing HCV treatment as well as those not currently accessing HCV treatment. Decisions to access treatment seemed to be strongly interlinked with agency, as well as reciprocity, as a number of inmates cited family members as their primary reason for undergoing treatment.

Because I didn’t want hep C. I wanted to be clean. I…I thought about my daughters. I thought about my partner. (Keith, 38, classification not recorded*)

Because…funny that…I’ve had it for so many years. […] But I want to be as supportive as I can for my kids and my family. I know I can’t help out a lot, but I’m doing something. (Jake, 47, A)

I’ve got a 14-year-old daughter, and I’ve got to start thinking about her future and that too. […] Doing this treatment will probably put another 10, 20 years to me life. So… [*So she’s an incentive for you to do the treatment?*] Yeah, she’s always been my incentive to do any good for me-self. (Lee, 33, B*)

Participants who expressed higher levels of agency often described trust and safety as being an important element in their personal pursuit of change. These participants described situations in which they felt another person had believed in them, particularly when they perceived that a staff member had gone ‘above and beyond’ to provide support, care, and consideration for these participants. In this regard, trust and safety was interlinked with agency whereby motivation for change was reliant on another’s belief in the individual.

Like I speak to the psych here, because the one that runs [one of the programs], like I trust her, because when they tried kicking me out before, they thought I was a security risk, she went in to bat for me, and that showed me that she thought I could change, you know? So I thought I’ll give her a chance. I started talking to her, and she’s alright. (Hugh, 39, C*)

The converse also appeared to hold, such that when inmates felt let down by a staff member, it impacted the participant’s sense of personal value and diminished their agency. Being treated as a “crim” rather than an individual appeared to negatively impact a participant’s agency and have the capacity to undermine the individual’s belief in self; if others couldn’t believe in them, how could they believe in themselves? This is reminiscent of the response to being treated as an individual, rather than a ‘crim’, with regards to feelings of trust and safety with personnel.

Um…I just felt like the system sort of let me down. If they didn’t care about me, why should I care? (Paul, 36, E)

Many inmates articulated agency through their drive and motivation in making decisions that had both immediate and long-term impacts on their lives. The drive behind this personal growth (or change) was often interlinked with family and loved ones on the outside. In some instances, drive or inner motivation was reflective of reciprocity as well as agency as participants described feeling indebted to these family members who have provided social and/or practical support to them. Some participants expressed negative impacts on their self-worth, and consequently their agency, when staff members were perceived to behave in ways that devalued the participant as a person.

### Civic engagement

If civic engagement is understood as having opportunities for input into our environments and circumstances, the prison environment is not, at a fundamental or practical level, conducive to opportunities for civic engagement. In this study, one distinct opportunity for civic engagement emerged: research participation.

[*Why did you want to do the research today? Why did you think you would participate?*] Just something to get, to help other people and that. Like, give ‘em a bit of encouragement to do the treatment and that. It’s something like, if you’ve got hep C it’s worth getting the treatment instead of letting it just ruin your life. (John, 22, C)

And yous are good. Yous a breath of fresh air. It’s good to, to um, break that cycle that we normally live in. You know what I mean? […] To have a real conversation. To be able to talk about, like your personal issues and that, you know? I’m very, very reserved and that, in around the pods and around the inmates and especially the officers, you know? I don’t tell them, I wouldn’t tell them about half of this… (Gary, 31, A)

By participating in research, inmates have the opportunity to discuss and describe their experiences on the inside, and, in some research, directly contribute to the recommendations of the research. For others, such as Gary, participating in research may also be an opportunity to be treated as a person and to “have a real conversation”. There are few other opportunities for prisoners to engage with non-prisoners and be treated as a person with the capacity to make valuable contributions. Research participation is ultimately an expression of self-choice – of choosing to participate, to contribute to collective knowledge, and to shape outcomes. Thus, participation in research is an interesting indicator of social capital and unanticipated in the prison setting. Consequently, it can be presumed that a number of study participants already possess higher levels of social capital than inmates who declined participation in this study. However, due to the fiscal incentive in this study (AUD$10), some of the participation interests may be countered by other motives. Alternatively, potential participants may have declined participation if research was perceived as a form of authority; the choice to not participate exerts personal choice in an institution governed by authoritative controls.

## Discussion

A number of dimensions of social capital transfer to the prison environment and contribute to an inmate’s overall social capital portfolio. However, not all dimensions translate into the prison setting in the ways in which they are experienced or accessed in the general population. Reliance on and resistance of those in authoritative positions was central to understanding the translation of trust and safety to the prison setting. Acknowledging inmates as a person, in any capacity – whether as a student or a patient – was a determinant of a staff member’s trustworthiness. Likewise, trust and safety among inmates carried burdens that were less prevalent in the community including a greater need to mask vulnerabilities for self-protection. Friendships among inmates appeared to have a distinct end-date as many participants confined friendships to the prison and did not maintain these relationships beyond release. An inmate’s informal networks on the outside, predominantly family and inclusive of some friends, emerged as a financial lifeline for many participants. Formal networks in prison were interlinked with trust and safety whereby inmates who were trusted by corrective services personnel were given valued employment positions. This had the benefit of improving an inmate’s employment situation, and, potentially, their financial situation within prison. Agency, the capacity and motivation to make decisions which impact one’s life, requires forward thinking. In prison, this dimension is most apparent in an inmate’s post-release planning, regardless of how distant their release date may be. Agency is indicative of health and wellbeing, reflective of self-action and a person’s decision to make change – key features in accessing rehabilitative or other health improvement programs. Civic engagement, a dimension which could be considered as significantly diminished within prison, does translate into the prison setting, for example through participation in research which was described as an opportunity for inmates to have a voice.

Translated into the prison setting, trust and safety can have far-reaching implications on inmates’ social capital. Trust and safety between inmates and staff (corrective services and health staff) displayed a mirrored pattern of stereotyping; an initial lack of treating the other as an individual, and instead, treating and thinking of the other in terms of what they represent (criminal or authority). From the inmate perspective, this seemed indicative of the ‘inmate code’ whereby *all* staff members were deemed untrustworthy. The inmate code is a social norm among inmates in which their behaviour is in direct contrast to the expectations of the prison administration (Wellford, 1967). For inmates adhering to the inmate code, disengagement with staff may also be indicative of social cohesion and trust among inmates.

Issues of trust can influence accessing healthcare – both in prison and general society (Altice et al. 2001; Howerton et al. 2007; Schwei et al. 2014). For injecting drug users, accessing healthcare in the community is complicated by the stigma of addiction (Treloar & Rance, 2014). In prison, trusting a health professional or officer appeared to be indicative of how the individual staff member treats the inmate, not simply the provision of health or other care, and is dependent on whether the practitioner or officer treats men in prison as individuals or ‘crims’.

The dimension of informal networks appeared to be both transferable and translatable to the prison setting and produce similar benefits for prisoner and community populations. Maintaining contacts with informal networks, such as loved ones, on the outside can improve the quality of life for those in prison, although these benefits are often gained at the costs of others. In a study pertaining to the social and economic capital of the families of incarcerated men, it was found that women on the outside often compromise their own quality of life in efforts to improve the quality of life of their loved one inside (Christian et al. 2006). The generosity was often provided in response to men’s promises of lifestyle changes upon the inmate’s return home. In another study, financial support from loved ones on the outside was shown to correspond with agency in a study pertaining to male inmates in the US (Leahy, 1998).

In prison, formal networks appeared to be closely interlinked with agency. Some inmates reported seeking out education in prison as a means of gaining qualifications to improve their circumstances upon release. However, inmates not engaged in formal networks may have limited opportunity to express or exert agency as they are less exposed to opportunities for encouragement and recognition of capability and achievements. It may be that the social capital gained through formal networks (such as education programs or employment) is not readily recognised, or lacks value, until the participant is released and there is greater scope for participation in the community. Thus, it seems that for men in prison, the utility derived from formal networks in prison provides greater benefits at a future time (such as rehabilitative programs which may reduce sentence length or education programs which provide qualifications for post-release employment), reflecting the inter-relationship with agency. Further research into the social capital benefits of formal networks among inmates should explore the value, or potential value, ascertained by inmates engaging in these formal networks.

Agency is both transferable and translatable to the prison setting, but with distinct variances to that described in the mainstream literature. The expression of agency among participants revolved around post-release planning and decisions around health, particularly accessing HCV treatment whilst in custody, both of which required elements of initiating action. However, expressions of agency seemed to be influenced by whether participants felt others believed in them, related to both formal and informal networks. Another unique component of agency in prison was its interconnectedness with reciprocity, adding to the complexity of motivations and capacity for initiating change. The forward-thinking indicative of post-release planning may be a coping mechanism of some inmates and allows them to feel connected to loved ones outside by thinking about a future with them.

Inmates who discussed post-release planning had varied sentence lengths, including those with over a decade remaining. This is in contrast to a study of women prisoners which found that women with longer sentences remaining experienced greater levels of powerlessness compared with women who had shorter time left to their sentences (Larson & Nelson, 1984). However, gender differences among the experiences of sentence length may exist for men and women and should be explored in relation to social capital.

Civic engagement is a dimension of social capital which is transferable to the prison setting, but does not appear to be translatable. The participants in this study highlighted the importance of engaging, and having opportunity to have a voice, to be listened to, and to be heard. Their expression of civic engagement was participation in research. This suggests that inmates are likely to engage with the outside world, to maintain a sense of citizenship, when provided with the opportunity to do so. This is similar to findings presented in a reflective paper, authored by a researcher (Bosworth) and four adult prisoners (inclusive of men and women) (Bosworth et al. 2005). In the collaborative paper, research participation provided opportunity for inmates to voice their experiences and be heard with respect and dignity.

### Limitations

The study had a number of limitations. Firstly, the study focused only on inmates who were HCV positive and were aware of their serostatus. Having consented to participate and undertake blood-borne virus (BBV) testing may in itself be an indication of higher levels of social capital, adjustment to prison, and other social and emotional wellbeing indicators. Also, inmates may be reluctant to test for BBVs for fear of being known by custodial authorities as being injecting drug users and, consequently, subject to greater surveillance. Inmates choosing to be tested may be more likely to be involved in drug treatment programs or less likely to be currently using drugs. However, further research is required to provide insight into such speculations. Participants were recruited through the health clinics which allowed for participants to maintain confidentiality of their serostatus within the prison (as we recruited through clinic staff with access to inmate health records). Secondly, the scope of this study did not allow for a full audit of the prison environment (Liebling, 2004) such as examining the relationship dynamics between inmates and staff, as well as between staff (including health and custodial staff), volunteers, and other personnel within the prison environment. The unique culture of each prison has the potential to greatly influence collaborative relationships between inmates as well as with personnel.

This study included only male participants and may not be applicable to the social capital of women in prison living with HCV. Incarcerated women have different needs than incarcerated men (Reisig et al. 2002) – their absence is likely to have greater impact on family and carer responsibilities (Burgess & Flynn, 2013) and they are likely to experience the double stigma of being a drug user (for those with a history of injecting drug use) and a mother (Radcliffe, 2011).

## Conclusion and implications

A number of social capital dimensions were shown to be transferable to the prison setting. While some of these dimensions were shown to require context-specific translation for the prison environment, many appeared to operate in a similar fashion to the community. Elements of social capital among prisoners may be influenced by structural factors such as prison culture, an inmate’s age, sentence length (including time served and proximity to release), and security classification. These relationships require additional investigation.

In particular, reciprocity emerged as an essential sub-theme across numerous social capital dimensions and was found to be integral to other social capital dimensions; in effect, reciprocity was not an individual component of social capital among men in prison but rather an overarching theme among many dimensions of social capital. In addition, reciprocity required translation for the prison setting from its use in mainstream populations. Reciprocity was often the underpinning motivation when expressing agency, such as reasons for accessing HCV treatment or post-release planning.

Trust may be a key social capital dimension in prison to consider in efforts to promote engagement in health services, education, and rehabilitative programs. These results show that trust can be established when inmates are treated as individuals; this development of trust may provide immediate and long-term benefits in improving healthcare and other agency-based outcomes (such as accessing education) among inmates. Although trust in prison shared commonalities with trust in the mainstream, the findings of this study show that building trust between inmates and corrective services personnel (including officers and healthcare workers) is achievable.

While prison is an ideal opportunity for rehabilitation and engagement with health and educational programs, high levels of social capital may be an important element of the initial access and engagement process (requiring agency for inmates to engage and participate as well as trust in the staff members delivering the program). Promoting health and other rehabilitative services as opportunities for inmates to contribute to the wellbeing of their families may produce greater uptake and adherence of treatment(s) in the prison environment. Provided that trust and agency are part of a person’s social capital ‘portfolio’ on the inside, improved access to rehabilitative programs and healthcare seem possible. Further research is needed to explore how social capital can be fostered and utilised in prison to increase participation in health services and other treatments, thus improving social and health outcomes for prisoners and their families.
